# Baicalein inhibits NLRP3 inflammasome activation and mitigates placental inflammation and oxidative stress in gestational diabetes mellitus

**DOI:** 10.1515/biol-2022-0966

**Published:** 2024-12-31

**Authors:** Jun Yao, Jiaying Pan, Qiaoying Jiang, Hui Wang, Yiqi Zhao

**Affiliations:** Center for Reproductive Medicine, Department of Obstetrics, Zhejiang Provincial People’s Hospital, Affiliated People’s Hospital, Hangzhou Medical College, No. 158 Shangtang Road, Hangzhou, Zhejiang, 310014, China; Department of Obstetrics and Gynecology, Xianju County People’s Hospital, Taizhou, Zhejiang, 317399, China

**Keywords:** Baicalein, gestational diabetes, NLRP3 inflammasome, inflammation, oxidative stress

## Abstract

Gestational diabetes mellitus (GDM) is a common metabolic disorder during pregnancy characterized by glucose intolerance, which poses risks to both maternal and fetal health. Baicalein, a flavonoid derived from the roots of *Scutellaria baicalensis* Georgi, exhibits various biological functions and has been implicated in the modulation of several diseases. However, the regulatory effects and underlying mechanisms of Baicalein in GDM progression remain unclear. In this study, we found that Baicalein ameliorates metabolic disturbances in GDM mice by improving glucose tolerance, insulin sensitivity, fasting blood glucose levels, and plasma insulin levels. Additionally, Baicalein treatment positively impacted litter size and birth weight. GDM mice exhibited increased inflammation and oxidative stress, which were mitigated following Baicalein administration (40 mg/kg). Furthermore, elevated protein levels of NLRP3, IL-1β, and IL-18 observed in GDM mice were reduced by Baicalein treatment. In conclusion, Baicalein inhibits the NLRP3 inflammasome and alleviates placental inflammation and oxidative stress associated with GDM. These findings provide valuable insights into the potential therapeutic role of Baicalein in managing GDM.

## 1 Introduction

Gestational diabetes mellitus (GDM) is a prevalent metabolic disorder that occurs during pregnancy, characterized by glucose intolerance that typically manifests in the second and third trimesters [1]. This condition is associated with several adverse outcomes, including abnormal embryonic development, insulin resistance, hyperinsulinemia, and hyperglycemia [2]. The incidence of GDM has been rising, primarily due to increasing maternal obesity and advanced maternal age [3]. GDM significantly increases the risk of severe complications for both mother and infant, such as macrosomia, dystocia, and neonatal hypoglycemia [4]. Despite ongoing research, the pathogenesis of GDM remains incompletely understood. Known etiological factors include oxidative stress, insulin resistance, and inflammation, which are triggered by placental hormones and strongly linked to the progression of GDM [5]. Therefore, the identification of novel and effective therapeutic agents is critical for managing GDM.

Baicalein is a flavonoid compound extracted from the roots of *Scutellaria baicalensis* Georgi, known for its diverse pharmacological effects [6]. Recent studies have revealed various biological functions of Baicalein, including its role in regulating different diseases. For instance, Baicalein can alleviate oxidative stress and promote autophagy, and was shown to improve cardiac hypertrophy in mice [7]. Additionally, Baicalein targets the GPX4/ACSL4/ACSL3 axis to inhibit ferroptosis, thereby reducing cerebral ischemia-reperfusion injury [8]. In hyperlipidemic pancreatitis, Baicalein modulates the miR-192-5p/TXNIP axis to inhibit the NLRP3/Caspase-1 pathway, thereby mitigating pyroptosis and inflammation [9]. Moreover, Baicalein regulates the AhR/IL-22 pathway to enhance the intestinal epithelial barrier, which alleviates ulcerative colitis [10]. Notably, Baicalein has been shown to target the miR-17-5p-Mfn1/2-NF-κB pathway in trophoblast cells, reducing high glucose-induced inflammation and apoptosis [11]. Despite these findings, the regulatory effects and mechanisms of Baicalein in GDM progression *in vivo* remain unexplored.

In this study, we demonstrate that Baicalein inhibits the NLRP3 inflammasome and alleviates placental inflammation and oxidative stress in a GDM mouse model, suggesting that Baicalein holds potential as a therapeutic agent for managing GDM.

## 2 Materials and methods

### 2.1 Animal model

C57BL/KsJ^+/+^ (wild-type) and C57BL/KsJ^db/+^ (db/+) mice (10 weeks old, *n* = 24 per sex) were obtained from the Nanjing Model Animal Center (Nanjing, China). All animal procedures were conducted in accordance with the guidelines of the Ethics Committee of Zhejiang Provincial People’s Hospital. Female mice were individually paired with male mice of the same genotype. The presence of copulatory plugs the following morning was used to confirm mating and designate gestation day (GD) 0. Pregnant female mice were then divided into four groups: Control (normal pregnancy, C57BL/KsJ^+/+^), GDM (C57BL/KsJ^db+^ (db/+) mice treated with DMSO), GDM + 20 mg/kg Baicalein (C57BL/KsJ^db+^ (db/+) mice treated with 20 mg/kg Baicalein), and GDM + 40 mg/kg Baicalein (C57BL/KsJ^db+^ (db/+) mice treated with 40 mg/kg Baicalein). Each group consisted of six mice. Baicalein (98% purity, S25956, Shanghai Yuanye Bio-Technology Co., Ltd, Shanghai, China) was dissolved in DMSO and administered orally at doses of 20 or 40 mg/kg daily for 20 days starting from pregnancy. On GD 10, glucose and insulin tolerance were assessed using intraperitoneal glucose tolerance tests (IPGTT) and intraperitoneal insulin tolerance tests (IPITT). On GD 20, fasting blood glucose levels were measured with a glucometer (Roche Diagnostics, Risch-Rotkreuz, Switzerland), and insulin levels were quantified using an Ultra-Sensitive Mouse Insulin ELISA kit (ALPCO Diagnostics, Salem, NH). Cesarean sections were performed on GD 20 to collect placentas and fetuses for further analysis. Litter size and birth weight were recorded.


**Ethical approval:** The research related to animal use has been complied with all the relevant national regulations and institutional policies for the care and use of animals, and has been approved by the Experimental Animal Welfare Ethics Committee of Zhejiang Provincial People’s Hospital (Approval no. 2021020).

### 2.2 Detection of glucose and insulin tolerance

For the IPGTT, after a 6 h fast, the mice received an intraperitoneal injection of glucose (2.0 g/kg). Blood glucose levels were measured at 0, 30, 60, 90, and 120 min using an ACCU-CHEK Advantage glucometer (Roche Diagnostics, Risch-Rotkreuz, Switzerland).

For the IPITT, after a 6 h fast, insulin (0.75 U/kg) was administered intraperitoneally. Blood glucose levels were then measured at 0, 30, 60, 90, and 120 min.

### 2.3 ELISA

The levels of TNF-α (ab208348, Abcam, Shanghai, China), IL-1β (ab197742), and IL-6 (ab222503) were quantified using commercial ELISA kits according to the manufacturer’s protocols.

### 2.4 Detection of oxidative stress

Oxidative stress markers, including malondialdehyde (MDA, ab118970, Abcam, Shanghai, China), glutathione (GSH, ab65322), myeloperoxidase (MPO, ab105136), and superoxide dismutase (SOD, ab65354), were measured using commercial kits in placental tissue samples.

### 2.5 Western blot

Proteins from mice placental tissues were extracted using RIPA lysis buffer (Thermo Fisher Scientific, Inc.), separated using 10% SDS-PAGE, and transferred to PVDF membranes (Beyotime, Shanghai, China). After blocking, membranes were incubated with primary antibodies at 4°C for 12 h. Secondary antibodies (1:2,000; ab7090) were then applied. Protein detection was performed using a chemiluminescence detection kit (Thermo Fisher Scientific, Inc.).

The primary antibodies used in this study were as follows: NLRP3 (1:1,000; ab263899; Abcam, Shanghai, China), IL-1β (1:1,000; ab283818), IL-18 (0.5 µg/mL; ab191860), and β-actin (1:2,000; ab8227).

### 2.6 Statistical analysis

Data are presented as mean value ± standard deviation (SD) and analyzed using GraphPad Prism 9 (GraphPad Inc, La Jolla, CA, USA). Comparisons were performed using one-way analysis of variance with Tukey’s *post hoc* test. A *p*-value < 0.05 was considered statistically significant.

## 3 Results

### 3.1 Baicalein improves metabolic symptoms in GDM mice

**Figure 1:**
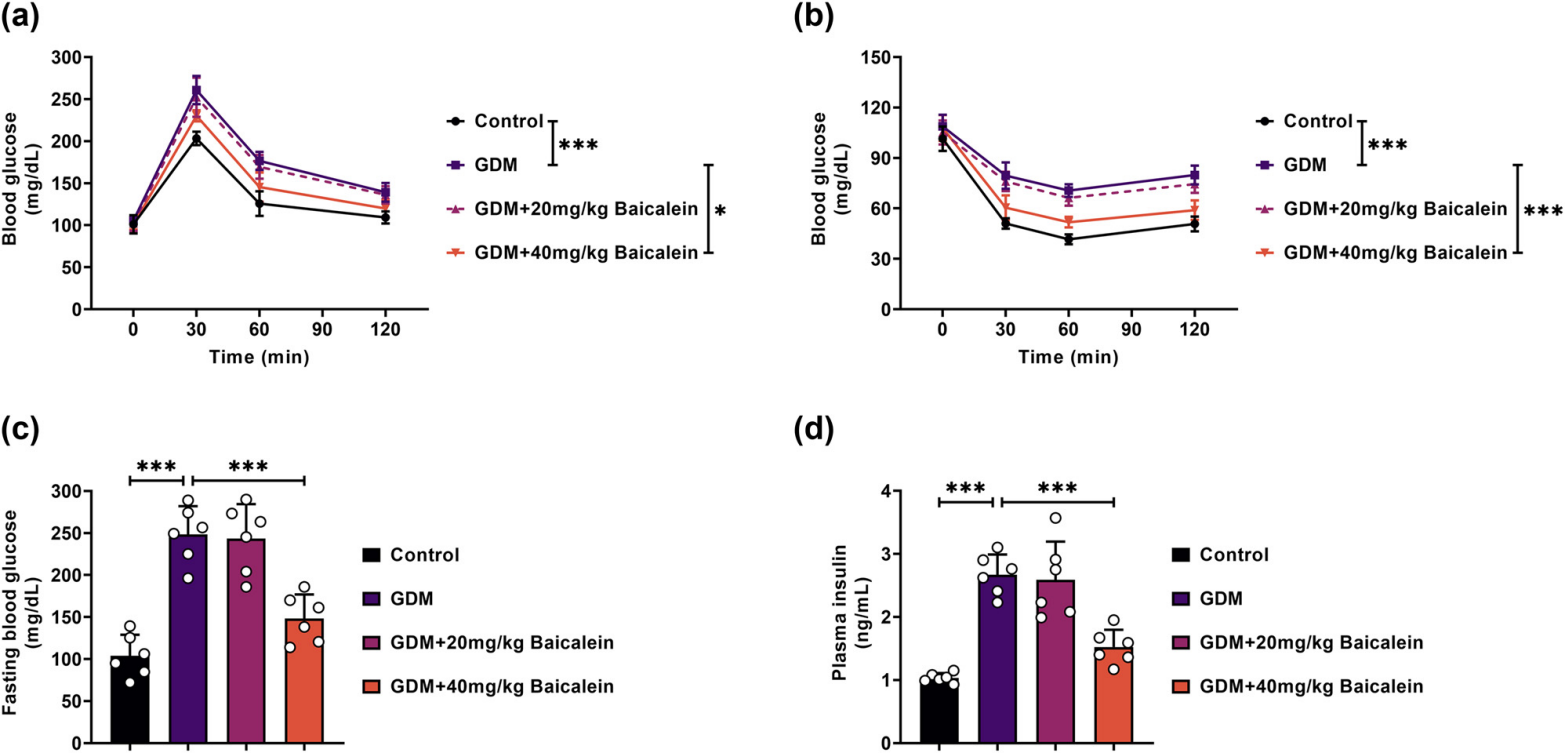
Baicalein ameliorates metabolic symptoms in GDM mice. The mice were divided into four groups: Control, GDM, GDM + 20 mg/kg Baicalein, and GDM + 40 mg/kg Baicalein. (a) Glucose tolerance was assessed using the IPGTT. (b) Insulin tolerance was evaluated via the IPITT. (c) Measurements of fasting blood glucose levels. (d) Assessment of plasma insulin levels. *p < 0.05, ***p < 0.001.

Our results showed that the glucose tolerance in GDM mice was impaired, but this impairment could be reversed using Baicalein (40 mg/kg) (Figure 1a). Similarly, insulin tolerance was reduced in GDM mice, and similarly, this reduction could be mitigated using Baicalein (40 mg/kg) (Figure 1b). Additionally, fasting blood glucose and plasma insulin levels were elevated in GDM mice, and these elevations could be normalized following Baicalein (40 mg/kg) treatment (Figure 1c and d). Overall, our findings indicate that Baicalein could improve metabolic symptoms in GDM mice.

### 3.2 Baicalein enhances reproductive outcomes in GDM mice

**Figure 2:**
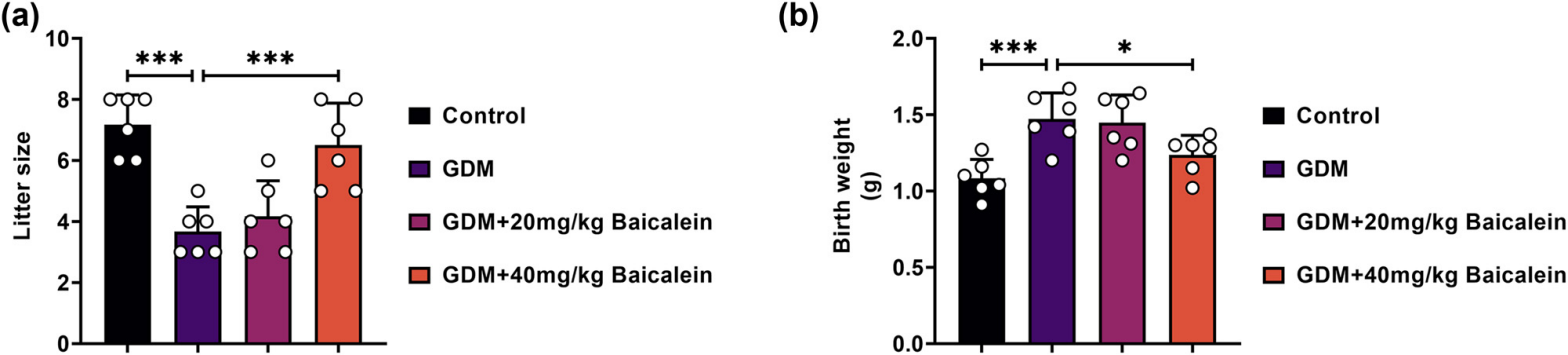
Baicalein improves litter size and birth weight in GDM mice. The mice were assigned to the following groups: Control, GDM, GDM + 20 mg/kg Baicalein, and GDM + 40 mg/kg Baicalein. (a) Recording of the litter size. (b) Measurements of birth weight. Statistical significance: *p < 0.05, ***p < 0.001.

In the GDM group, the number of offspring per litter was reduced, but this reduction was alleviated by Baicalein (40 mg/kg) treatment (Figure 2a). Furthermore, the birth weight of the offspring was increased in the GDM group; this effect was attenuated with Baicalein (40 mg/kg) administration (Figure 2b). Thus, Baicalein positively influenced reproductive outcomes in GDM mice.

### 3.3 Baicalein reduces inflammation and oxidative stress in GDM mice

**Figure 3:**
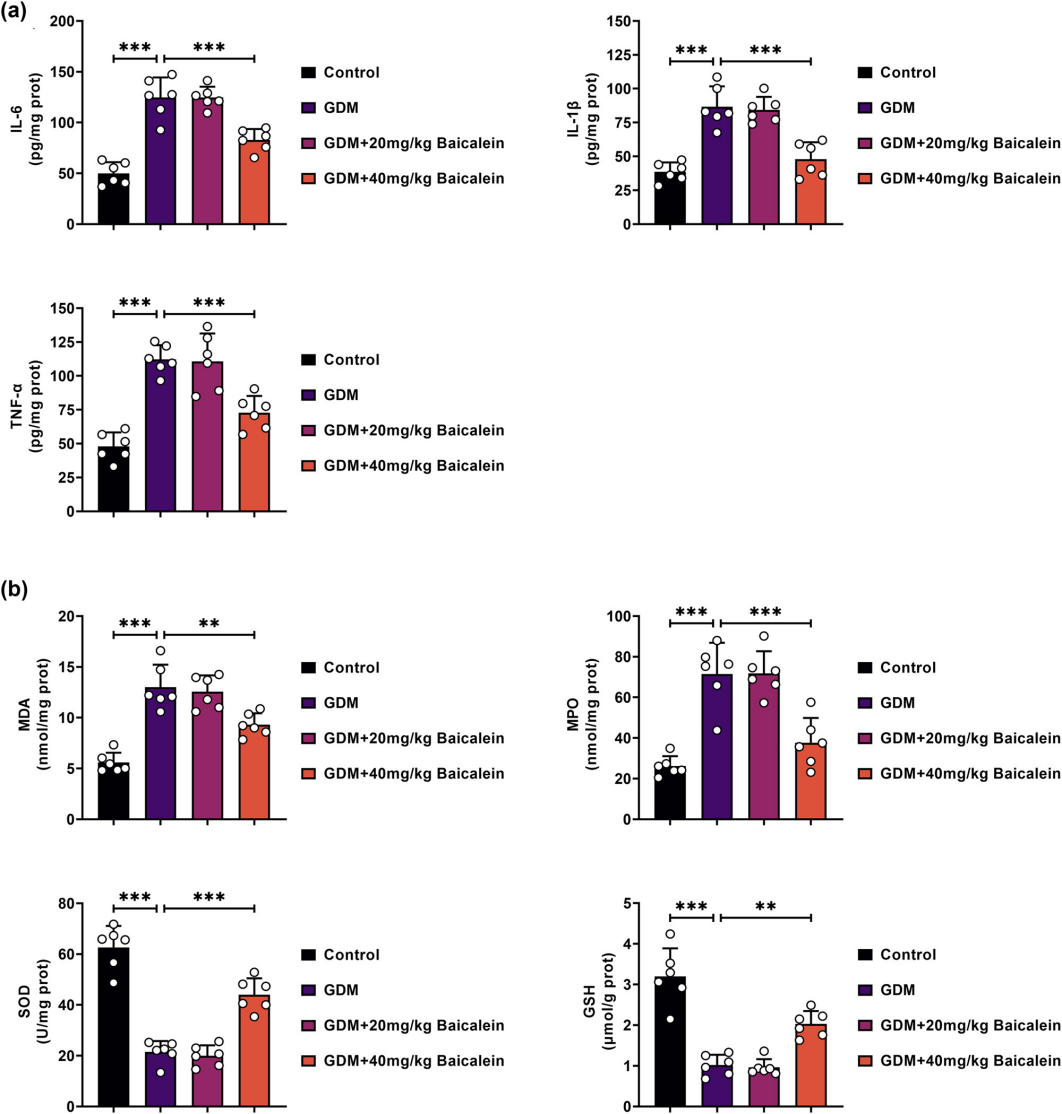
Baicalein reduces inflammation and oxidative stress in GDM mice. The mice were categorized into the Control, GDM, GDM + 20 mg/kg Baicalein, and GDM + 40 mg/kg Baicalein groups. (a) Levels of IL-6, IL-1β, and TNF-α were quantified using ELISA. (b) The levels of MDA, SOD, MPO, and GSH were assessed with commercial kits. Statistical significance: **p < 0.01, ***p < 0.001.

ELISA results showed that levels of IL-6, IL-1β, and TNF-α were elevated in GDM mice, but these increases were significantly reduced following Baicalein (40 mg/kg) treatment (Figure 3a). Additionally, markers of oxidative stress, including MDA and MPO, were elevated, while SOD and GSH levels were decreased in GDM mice. Baicalein (40 mg/kg) treatment normalized these oxidative stress markers (Figure 3b). Taken together, these findings indicate that Baicalein could effectively alleviate inflammation and oxidative stress in GDM mice.

### 3.4 Baicalein attenuates NLRP3 inflammasome activation in placentae of GDM mice

**Figure 4:**
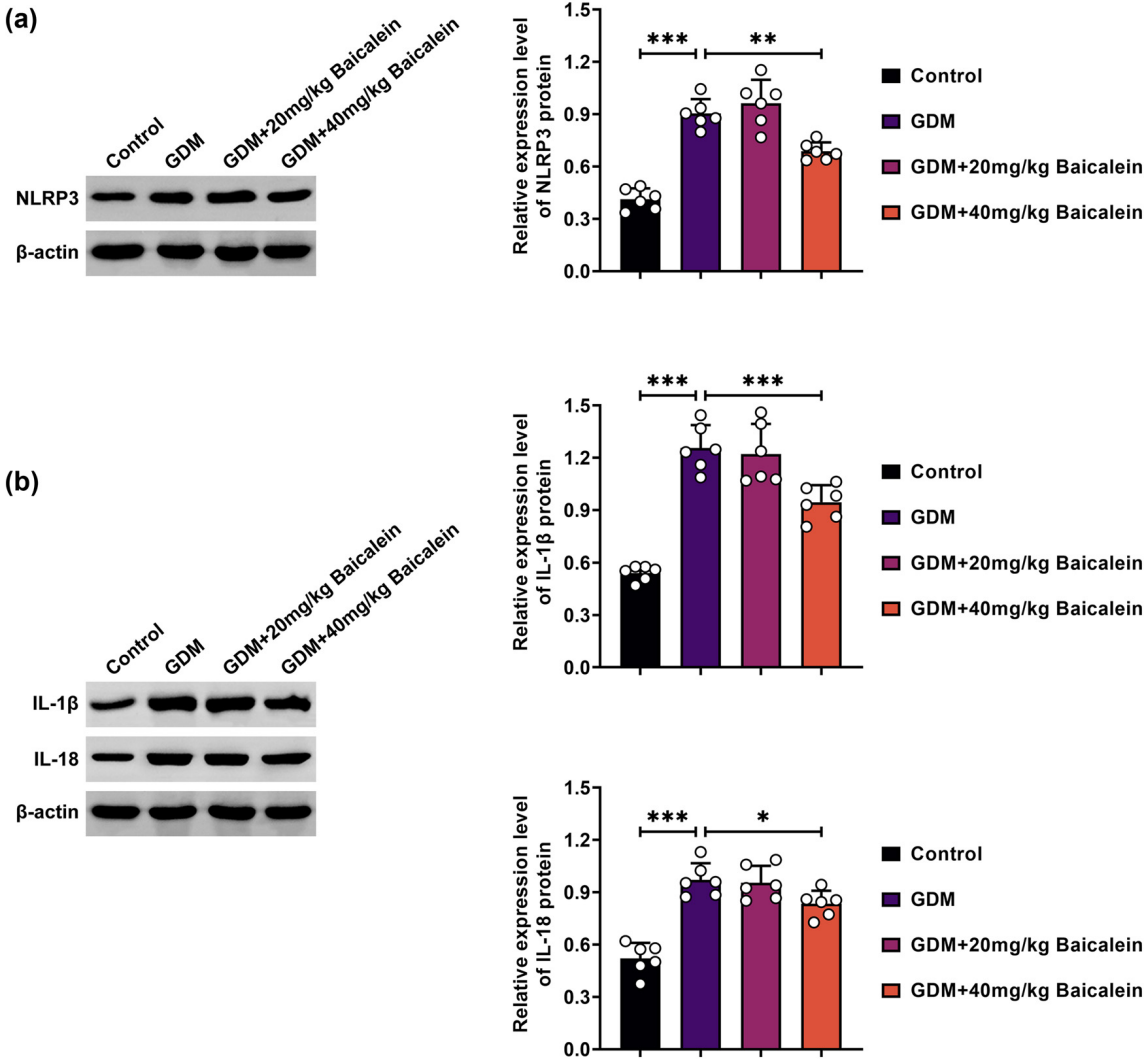
Baicalein attenuates placental NLRP3 inflammasome activation in GDM mice. The mice were grouped into Control, GDM, GDM + 20 mg/kg Baicalein, and GDM + 40 mg/kg Baicalein. (a) NLRP3 protein expression was analyzed by Western blot. (b) Protein levels of IL-1β and IL-18 were also assessed by Western blot. Statistical significance: *p < 0.05, **p < 0.01, ***p < 0.001.

NLRP3 protein expression was elevated in the placentae of GDM mice, but this elevation was reduced following Baicalein (40 mg/kg) treatment (Figure 4a). Similarly, levels of IL-1β and IL-18 were increased in GDM mice, but these increases were diminished after Baicalein (40 mg/kg) administration (Figure 4b). Collectively, Baicalein decreased NLRP3 inflammasome activation in the placentae of GDM mice.

## 4 Discussion

Numerous extracts from Chinese herbs have been shown to influence the progression of gestational diabetes mellitus (GDM). For instance, Polyphyllin I modulates the AMPK pathway to reduce inflammatory damage in GDM [12]. Similarly, Astragaloside IV alleviates placental inflammation and oxidative stress in GDM mice [13], and Resveratrol activates the AMPK pathway to improve GDM progression in mice [14]. Baicalein, a flavonoid with diverse biological functions, has been implicated in the management of various diseases [7,8,9,10]. However, its regulatory roles and mechanisms in the context of GDM have not been fully elucidated. This study demonstrates that Baicalein improves metabolic symptoms in GDM mice by enhancing glucose and insulin tolerance, and normalizing fasting blood glucose and plasma insulin levels. Additionally, Baicalein positively affected reproductive outcomes, including the number of offspring per litter and birth weight.

Inflammation and oxidative stress play pivotal roles in the progression of GDM [15]. Consequently, many researchers have concentrated on modulating these factors to slow the progression of GDM. For instance, cryptotanshinone has been shown to alleviate placental inflammation and oxidative stress in GDM mice [16]. Similarly, N-acetyl-L-cysteine helps reduce both inflammation and oxidative stress associated with GDM [17]. Additionally, miR-875-5p targets TXNRD1, thereby influencing oxidative stress and inflammation in GDM rats [18]. Myrtenol has also been demonstrated to modulate the TLR4/MyD88/NF-κB pathway, thereby mitigating inflammation and oxidative stress in GDM rats [19]. In alignment with these findings, our study observed that inflammation and oxidative stress were elevated in GDM mice. However, these effects were significantly mitigated following treatment with Baicalein (40 mg/kg), suggesting that Baicalein can also effectively alleviate inflammation and oxidative stress in the context of GDM.

Inflammatory factors such as TNF-α, IL-6, and C-reactive protein are known to contribute to insulin resistance in GDM [20]. The nucleotide-binding oligomerization domain-like receptor thermal protein domain associated protein 3 (NLRP3) inflammasome, a macromolecular complex consisting of NLRP3, caspase-1, and ASC, plays a critical role in this process [21]. The interaction between caspase-1 and pathogen-associated molecular patterns or damage-associated molecular patterns activates the NLRP3 inflammasome, leading to the secretion of IL-1β and IL-18 from immune cells and promoting inflammation [22]. Previous studies have shown that Baicalein can target the miR-17-5p-Mfn1/2-NF-κB pathway in trophoblast cells, reducing high glucose-induced inflammation and apoptosis [11]. Despite these findings, the effects of Baicalein on the NLRP3 inflammasome in the context of GDM progression *in vivo* have not been well characterized. In this study, we observed that protein levels of NLRP3, IL-1β, and IL-18 were increased in GDM mice. However, these effects were attenuated following Baicalein treatment, indicating that Baicalein inhibits the NLRP3 inflammasome.

In conclusion, Baicalein effectively inhibited the NLRP3 inflammasome and mitigated placental inflammation and oxidative stress associated with GDM in mice. Nevertheless, this study has certain limitations, including the need for further investigation using human samples, additional cell models, and a broader range of phenotypes. Future research should focus on these areas to provide a more comprehensive understanding of Baicalein’s effects and potential therapeutic applications.
